# Challenges and Costs of Asexuality: Variation in Premeiotic Genome Duplication in Gynogenetic Hybrids from *Cobitis taenia* Complex

**DOI:** 10.3390/ijms222212117

**Published:** 2021-11-09

**Authors:** Dmitrij Dedukh, Anatolie Marta, Karel Janko

**Affiliations:** 1 Laboratory of Fish Genetics, Institute of Animal Physiology and Genetics of the CAS, Rumburská 89, 277 21 Liběchov, Czech Republic; anatolmarta@gmail.com; 2 Department of Zoology, Faculty of Science, Charles University in Prague, 128 00 Prague, Czech Republic; 3 Institute of Zoology, MD-2028, Academiei 1, 2001 Chisinau, Moldova; 4 Department of Biology and Ecology, Faculty of Science, University of Ostrava, Chittussiho 10, 710 00 Ostrava, Czech Republic

**Keywords:** gynogenesis, meiosis, hybrid sterility, endoreplication, polyploidy, *Cobitis taenia* complex

## Abstract

The transition from sexual reproduction to asexuality is often triggered by hybridization. The gametogenesis of many hybrid asexuals involves premeiotic genome endoreplication leading to bypass hybrid sterility and forming clonal gametes. However, it is still not clear when endoreplication occurs, how many gonial cells it affects and whether its rate differs among clonal lineages. Here, we investigated meiotic and premeiotic cells of diploid and triploid hybrids of spined loaches (Cypriniformes: *Cobitis*) that reproduce by gynogenesis. We found that in naturally and experimentally produced F1 hybrids asexuality is achieved by genome endoreplication, which occurs in gonocytes just before entering meiosis or, rarely, one or a few divisions before meiosis. However, genome endoreplication was observed only in a minor fraction of the hybrid’s gonocytes, while the vast majority of gonocytes were unable to duplicate their genomes and consequently could not proceed beyond pachytene due to defects in bivalent formation. We also noted that the rate of endoreplication was significantly higher among gonocytes of hybrids from natural clones than of experimentally produced F1 hybrids. Thus, asexuality and hybrid sterility are intimately related phenomena and the transition from sexual reproduction to asexuality must overcome significant problems with genome incompatibilities with a possible impact on reproductive potential.

## 1. Introduction

Species are fundamental evolutionary units, presumably evolving in a continuum from intermixing populations to independent entities isolated from other species by pre- and postzygotic barriers [1,2]. Their formation is thus frequently accompanied by interspecific hybridization, which may have positive [1,3,4,5] as well as negative impacts [1,6,7] and appears to be a mighty evolutionary force. With incompatibilities accumulating among their genomes, the crossing of parental species often affects the fertility of hybrids ultimately leading to their sterility [8,9]. Hybrid sterility may have various causes [10,11] and its molecular underpinning is still little understood. However, an important cause of hybrid sterility is the improper pairing and recombination between orthologous chromosomes from two different parental species during meiotic prophase, leading to the abruption of meiosis and/or the formation of aneuploid gametes [1,6,7]. Nonetheless, hybridization not only affects interactions between the two admixed genomes but it can also modify gametogenic pathways and induce the switch of a hybrid’s reproduction towards asexuality [5,12,13,14,15,16].

Traditionally, sex and asexuality have been viewed as contrasting dichotomies, but in reality, they rather represent two extremes of a continuum. Indeed, even sexual organisms can sometimes spontaneously produce unreduced gametes [17,18,19] and asexual organisms in fact represent a very diverse group that employs a wide spectrum of cytological mechanisms for gamete production. These range from completely ameiotic processes (apomixis) to those involving more or less distorted meiotic divisions (automixis) [15,20,21,22]. Surprisingly, although sexual and asexual reproduction represent a major and intensively studied paradox of evolutionary biology [2,23,24], very little is known about the cellular and molecular machinery causing the alterations between both reproduction types. Detailed investigation of these pathways could give an answer to basic questions such as what mechanisms cause the transitions from sexual reproduction to asexuality; why are some types of gametogenic aberrations are more common than others; and, what challenges do asexuals face during the alterations of cellular pathways?

**Figure 1:**
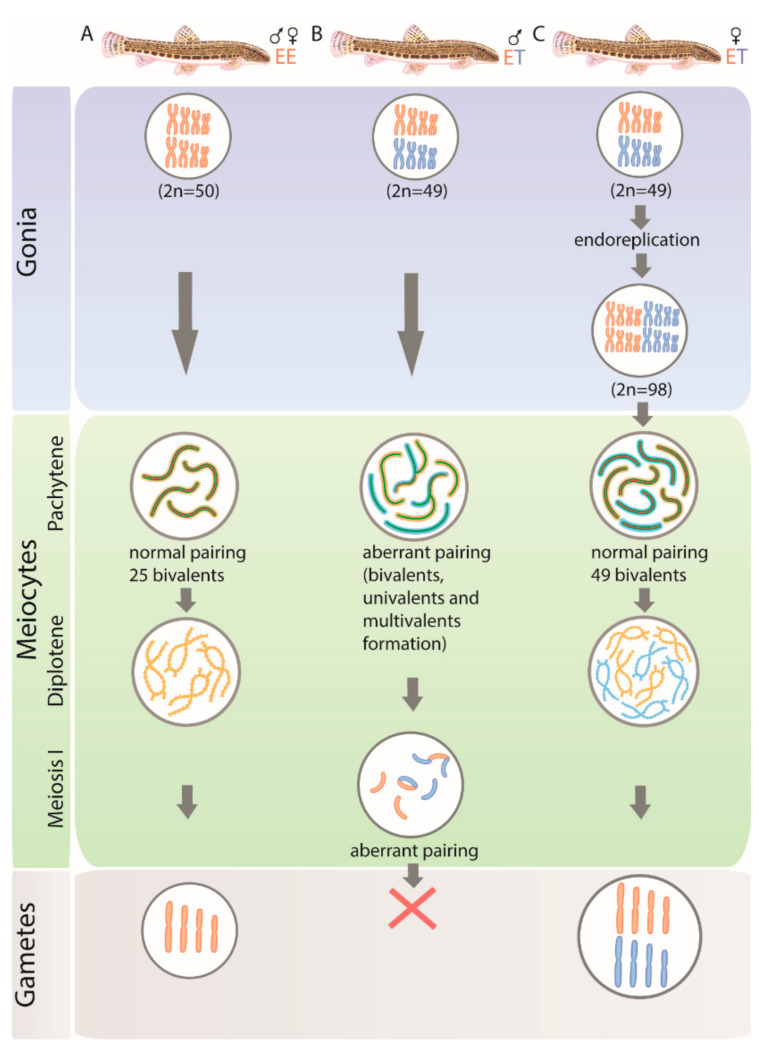
Schematic overview of gametogenic pathway during sexual reproduction (**A**), hybrid sterility (**B**) and asexuality (**C**). Each column shows individuals, gametogenic pathways with the indication of the germ cells, cells in two meiotic stages (pachytene and diplotene) and gametes. (**A**) (Orange chromosomes), (**B**) (blue chromosomes) indicates genomes of different parental species, *C. elongatoides* and *C. taenia*, correspondingly. (**A**) During sexual reproduction, gonia cells enter meiosis, which results in a haploid egg. Homologous chromosomes are joined by the synaptonemal complex (green color indicates the lateral element of synaptonemal complexes; red color indicates the central element of synaptonemal complexes) during pachytene, which is disassembled by the diplotene stage of meiosis. (**B**) Hybrid sterility caused by aberrant chromosome pairing during pachytene (also see [25]). (**C**) Reproduction of asexual hybrids is realized by endoreplication of the genomes in gonial cells allowing bivalent formation between identical chromosomal copies resulting in diploid gamete formation (modified from [25]).

At least in hybrid asexuals, it appears that the switch from sexual to clonal reproduction involves the modification of conservative gametogenic pathways, which is already occurring in the F1 generation [15,16,20,21,22]. Interestingly, in cases where hybridization leads to aberrant chromosomal pairing and hybrid sterility, the adoption of clonal gametogenesis may partially overcome such conflicts and restore fertility (Figure 1B) [15,21,22,25,26]. One example of this is premeiotic endoreplication, which is a widespread gametogenic alteration found among a vast variety of unrelated asexual organisms such as plants, invertebrates and vertebrates [20,22,27,28]. During this process chromosomes of gonial cells are duplicated ensuring successful progression through meiosis due to bivalents forming between identical copies of chromosomes [26,29,30,31,32]. As a result, endoreplication rescues the hybrid’s fertility and ensures clonal propagation of the maternal genome (Figure 1C) [26,33]. Hybrid asexuality and sterility thus share some common cytological ground; both tend to emerge in hybrids between substantially diverged species rather than between closely related ones [12,25,34,35,36].

In-depth studies of asexual gametogenesis are relatively rare but it has been hypothesized that once a successful clone emerges most of its germ cells follow the same pathway towards production of unreduced gametes [35]. Surprisingly though, studies on laboratory synthesized hybrids between medaka fish species (*Oryzias latipes* × *O. curvinotus*) as well as on parthenogenetic lizards from genus *Aspidoscelis* both showed that endoreplication occurred only in a small portion of hybrid’s germ cells, while most of their oogonia failed to endoreplicate their genome and did not develop to gametes [37,38,39].

In this study, we aim to analyze the gametogenesis of natural clonal lineages as well as laboratory-induced hybrids in order to test whether premeiotic genome endoreplication is indeed occurring in all or the majority of the hybrid’s germ cells and if even successful natural clones face problems in gamete production inherent to their hybrid origin.

We focused on spined loaches *Cobitis* (Cypriniformes: Cobitidae), which are an excellent model with which to investigate the emergence and evolutionary consequences of asexuality. Three species of these freshwater fishes meet and reproductively interact in Europe: *C. taenia* with diploid karyotype involving 2*n* = 48 chromosomes in somatic tissues (henceforth its genome will be ‘T’, so that diploid pure species is denoted ‘TT’), *C. elongatoides* (EE, 2*n* = 50) and *C. tanaitica* (NN, 2*n* = 50) [40,41,42,43]. Previous studies showed that their hybridization produces hybrids both in laboratory and natural conditions [14,25]. Male hybrids between *C. elongatoides* and *C. taenia* are sterile due to the aberrant pairing of their chromosomes (Figure 1) [26,44]. Regardless of this, the fertility of the hybrid females is sustained by premeiotic genome endoreplication and consequently, diploid females (of ET or EN genetic constitution with 49 and 50 chromosomes, respectively) reproduce gynogenetically by clonal eggs [26,45]. Occasionally their eggs incorporate sperm from parental species leading to the establishment of triploid *C. elongatoides*-*taenia* (ETT, 3*n* = 73) and *C. elongatoides-tanaitica* (EEN, 3*n* = 75) gynogenetic females [26,40,42,43]. The hybridization among these species has been dynamic since the Pleistocene and has led to high clonal diversity. Many clones originated relatively recently during the Holocene and occur in secondary hybrid zones between *C. elongatoides* and *C. taenia* or *C. tanaitica*, but one successful clonal lineage, EEN, colonized vast areas over Europe since its ancient origin approximately 300 kya [46,47].

Here, we analyzed the gametogenic pathways of both experimental F1 and naturally occurring clonal lineages and tested the proportion of their cells that successfully develop into clonal gametes. Specifically, we focused on oocytes during the pachytene and diplotene as well as gonocytes and analyzed their distribution throughout ovaria of asexual hybrid biotypes, including diploid, triploid, newly synthesized F1s as well as successfully established natural clonal lineages. We also estimated the ploidy of individual germ and meiotic cells using species polymorphic and chromosome-specific probes using fluorescent in situ hybridization (FISH). In each type of hybrid, we investigated how its cells passed through meiotic checkpoints and checked the ability of their germ cells to undergo premeiotic duplication of the genomes leading to viable gametes.

## 2. Materials and Methods

### 2.1. Samples Studied and Crossing Experiments

Genome composition and ploidy of every investigated specimen were evaluated with the set of species-diagnostic markers including microsatellites, allozymes and cytogenetic methods [41]. In total, we analyzed 9 triploid ETT hybrid females, 4 triploid EEN hybrid females and 11 diploid hybrid females from nature localities and laboratory crosses. Two individuals of parental species (*C. elongatoides*) were used as a control. Any treatment or injection was used before the investigation of female gametogenesis. Animals were anesthetized in MS222 and sacrificed accordingly. Gonads of each individual were separated into several pieces followed by usage for pachytene and/or diplotene chromosome preparation and for observation under laser scanning confocal microscope. Gonadal tissues used for 3D analysis were fixed in 2% paraformaldehyde (PFA) in 1× PBS (4.3 mM Na_2_HPO_4_, 1.47 mM KH_2_PO_4_, 2.7 mM KCl, 137 mM NaCl, pH 7.4) for 90 min at room temperature (RT), washed in 1× PBS, and kept in 1× PBS with 0.02% NaN_3_ until usage.

Spawning was performed semi-naturally by placing pairs of selected individuals in separated aquariums with spawning boxes. After spawning parental individuals were removed from aquariums. Two to three months after hatching we randomly selected juveniles for the analysis of pachytene oocytes. Diplotene oocytes were analyzed from adult and subadult females older than half a year.

### 2.2. Pachytene Chromosomes and Immunofluorescent Staining

Pachytene chromosomes were obtained according to the protocol described by [48]. After manual homogenization of female gonads, 20 µL of cell suspension was dropped on SuperFrost^®^ slides (Menzel Gläser, Thermo Scientific, Saarbrücken, Germany) followed by the addition of 40 µL of 0.2 M sucrose and 40 µL of 0.2% Triton X100 for 7 min. Afterward, cells were fixed for 16 min by adding 400 µL of 2% PFA. After washing in 1× PBS slides were stored until immunofluorescent staining of synaptonemal complexes (SCs) was performed.

Lateral components of synaptonemal complexes (SCs) were detected by rabbit polyclonal antibodies (ab14206, Abcam, Cambridge, UK) against SYCP3 protein while the central component of SC was detected by chicken polyclonal antibodies against SYCP1 protein (gift from Prof. Sean Burgess; [49]). According to previously published data SYCP3 is localized on both on bivalents and univalents while SYCP1 indicates pairing and bivalent formation [49]. Fresh slides were incubated with 1% blocking reagent (Roche, Mannheim, Germany) in 1× PBS and 0.01% Tween-20 for 20 min followed by the addition of primary antibodies for 1 h at room temperature (RT). Slides were washed 3 times in 1× PBS at RT and incubated in a combination of secondary antibodies (Cy3-conjugated goat anti-chicken IgG (H + L) ( Invitrogen, San Diego, CA, USA) and Alexa-488-conjugated goat anti-rabbit IgG (H + L) (Invitrogen, San Diego, CA, USA) diluted in 1% blocking reagent (Roche) on 1× PBS for 1 h at RT. Slides were washed in 1× PBS and mounted in Vectashield/DAPI (1.5 mg/mL) (Vector, Burlingame, CA, USA).

### 2.3. Diplotene Chromosomes

Diplotene chromosomal spreads (so-called “lampbrush chromosomes”) were prepared from parental and hybrid females according to an earlier published protocol [50]. Ovaries from non-stimulated females were dissected and placed in the OR2 saline (82.5 mM NaCl, 2.5 mM KCl, 1 mM MgCl_2_, 1 mM CaCl_2_, 1 mM Na_2_HPO_4_, 5 mM HEPES (4-(2-hydroxyethyl)-1-piperazineethanesulfonic acid); pH 7.4). Oocyte nuclei were isolated manually using jeweler forceps (FST, Heidelberg, Germany) in the isolation medium “5:1” (83 mM KCl, 17 mM NaCl, 6.5 mM Na_2_HPO_4_, 3.5 mM KH_2_PO_4_, 1 mM MgCl_2_, 1 mM DTT (dithiothreitol); pH 7.0–7.2). Oocyte nuclei were transferred to glass chambers attached to a slide filled with one-fourth strength “5:1” medium with the addition of 0.1% paraformaldehyde and 0.01% 1 M MgCl_2_. Such a method ensures that each chamber contained chromosomal spread from the individual oocyte. The slide was subsequently centrifuged for 20 min at +4 °C, 4000 rpm, fixed for 30 min in 2% paraformaldehyde in 1× PBS, and post-fixed in 70% ethanol overnight (at +4 °C).

### 2.4. Fluorescence In Situ Hybridization

Probes for FISH procedures were selected according to earlier published data [43]. We selected chromosome-specific markers (satCE01) and centromeric repeat (satCE04), as well as a species polymorphic marker (satCE02) between *C. elongatoides* and *C. taenia*. Biotin and digoxigenin labeling of probes were performed by PCR using genomic DNA of *C. elongatoides* according to [43].

The hybridization mixture contained 50% formamide, 10% dextran sulfate, 2× ЅЅС, 5 ng/μL labeled probe, and 10–50-fold excess of tRNA. After common denaturation of the probe and chromosomal DNA on slides at 75 °C for five minutes, slides were incubated for 12–24 h at RT. After hybridization, slides were washed three times in 0.2× SSC at +44 °C. The biotin-dUTP and digoxigenin-dUTP were detected using streptavidin-Alexa 488 (Invitrogen, San Diego, CA, USA) and anti-digoxigenin-rhodopsin (Invitrogen, San Diego, CA, USA), respectively. The chromosomes were counterstained with Vectashield/DAPI (1.5 mg/mL) (Vector, Burlingame, CA, USA).

### 2.5. Whole-Mount Immunofluorescence Staining

Whole-mount immunofluorescent staining was performed according to the previously published protocol [32]. Prior to immunofluorescent staining, gonadal fragments were permeabilized in a 0.5% solution of Triton X100 in 1× PBS for 4–5 h at RT followed by washing in 1× PBS at RT. The following primary antibodies were used: rabbit polyclonal antibodies DDX4 antibody (C1C3, GeneTex Inc., Irvine, CA, USA) against Vasa protein; rabbit polyclonal antibodies against SYCP3 (ab14206, Abcam, Cambridge, UK); chicken polyclonal antibodies against SYCP1 protein (gift from Prof. Sean Burgess; [48]). After incubation for 1–2 h in a 1% blocking solution (Roche, Mannheim, Germany) dissolved in 1× PBS, primary antibodies were added for 12 h at RT. Secondary anti-rabbit antibodies conjugated with Alexa-488 fluorochrome and anti-chicken antibodies conjugated with Alexa 594 were applied for 12 h at RT. Washings from primary and secondary antibodies were carried out in 1× PBS with 0.01% Tween (ICN Biomedical Inc., Solon, USA). Tissues were stained with DAPI (1 mg/mL) (Sigma Aldrich, Saint Louis, USA) in 1× PBS at RT overnight.

### 2.6. Whole-Mount Fluorescence In Situ Hybridization

Whole-mount FISH was performed according to [32]. Briefly, gonadal fragments were permeabilized in a 0.5% solution of Triton X100 (Sigma Aldrich, Saint Louis, MO, USA) in 1× PBS for 4–5 h at RT followed by impregnation by 50% formamide, 10% dextran sulfate, and 2× SSC for 3–4 h at 37 °C. Afterward, tissues were placed in a hybridization mixture including 50% formamide, 2× SSC, and 10% dextran sulfate, 20 ng/µL probe, and 10 to 50-fold excess of salmon sperm DNA. Gonadal tissues were denaturated at 82 °C for 15 min and incubated for 24 h at RT. Tissues were washed in three changes of 0.2× SSC at 44 °C for 15 min each and subsequently incubated in 4 × SSC containing 1% blocking reagent (Roche) for 1 h at RT. The biotin-dUTP and digoxigenin-dUTP were detected using streptavidin-Alexa 488 (Invitrogen, San Diego, CA, USA) and anti-digoxigenin-rhodopsin (Invitrogen, San Diego, CA, USA), respectively. The tissues were stained with DAPI (1 mg/mL) (Sigma Aldrich, Saint Louis, MO, USA) diluted in 1× PBS at RT overnight.

### 2.7. Confocal Laser Scanning Microscopy

Tissue fragments were placed in a drop of DABCO antifade solution containing 1 mg/mL DAPI. Confocal laser scanning microscopy was carried out using a Leica TCS SP5 microscope based on the inverted microscope Leica DMI 6000 CS (Leica Microsystems, Wetzlar, Germany). Specimens were analyzed using HC PL APO 40× objective (Leica Microsystems, Wetzlar, Germany). Diode, argon and helium-neon lasers were used to excite the fluorescent dyes DAPI, fluorochromes Alexa488, Cy3 and rhodopsin, respectively. The images were captured and processed using LAS AF software (Leica Microsystems, Wetzlar, Germany).

### 2.8. Wide-Field and Fluorescence Microscopy

Meiotic chromosomes after FISH and immunofluorescent staining were analyzed using Provis AX70 Olympus microscope (Olympus Corporation, Tokyo, Japan) equipped with standard fluorescence filter sets. Microphotographs of chromosomes were captured by CCD camera (DP30W Olympus) (Olympus Corporation, Tokyo, Japan) using Olympus Acquisition Software (DP controller, 2.1.1.183, Olympus Corporation, Tokyo, Japan). Microphotographs were finally adjusted and arranged in Adobe Photoshop CS3 V. 10.0 (Adobe) software; Adobe Illustrator CC2020 V. 25.0 (Adobe) were used for scheme drawing. Imaris V. 7.7.0 (Bitplane, Oxford Instruments, Zurich, Switzerland) software was used for the 3D-volume and surface reconstruction of confocal image stacks. The image stacks used for reconstruction were cropped to the region of interest and used for the reconstruction of isosurfaces. The following channels were used to construct isosurfaces separately: DAPI channel and rhodopsin channel. Threshold parameters were selected automatically. To highlight the visualization of germ cells, only surface objects belonging to individual germ cells were retained in the reconstruction.

## 3. Results

### 3.1. All Diplotene Oocytes Have a Duplicated Genome and Properly Paired Chromosomes with Bivalents

Somatic cells of diploid ET hybrids have 2n = 49 chromosomes, while triploid hybrids with ETT and EEN genome composition have 3n = 73 and 75 chromosomes, respectively [40,42,43]. Consequently, after endoreplication, their germ cells should have 98, 146 and 150 chromosomes, respectively, leading to 49, 73 and 75 bivalents in oocytes of diploid ET and triploid ETT and EEN hybrids (this study; [26]). To test if the germ cells of diploid (natural and laboratory generated F1 hybrids) as well as triploid hybrids showed evidence of genome duplication, we determined the number of bivalents in oocytes during the diplotene stage.

**Figure 2:**
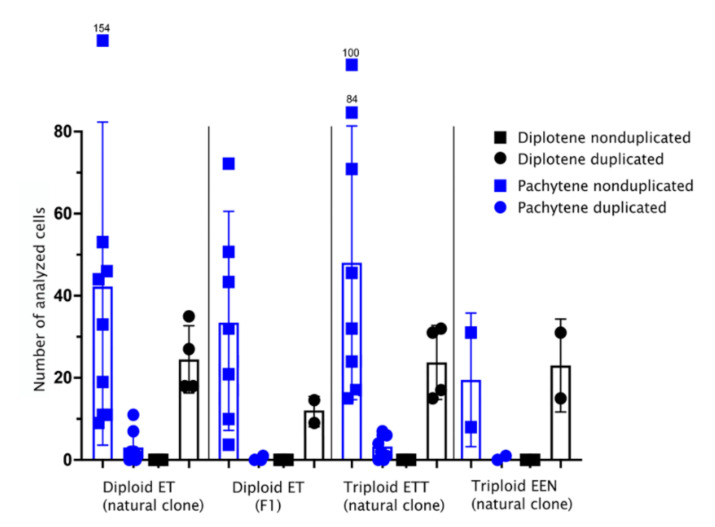
The number of cells with duplicated (marked as circles) and nonduplicated (marked as squares) genomes observed during pachytene and diplotene in natural diploid ET hybrids, artificial F1 diploid hybrids, and natural triploid ETT and EEN hybrids. The number of cells: mean ± standard deviation. During the pachytene stage (blue graphs), two populations of oocytes were detected in all diploid and triploid hybrid individuals. The major population of oocytes did not have a duplicated genome (oocytes with diploid and triploid genomes for diploid and triploid hybrids, correspondingly) and the minor population had a duplicated genome (oocytes with tetraploid and hexaploid genomes for diploid and triploid hybrids, respectively). During diplotene (black graphs), only cells with duplicated genomes were observed for all diploid and triploid hybrids.

**Figure 3:**
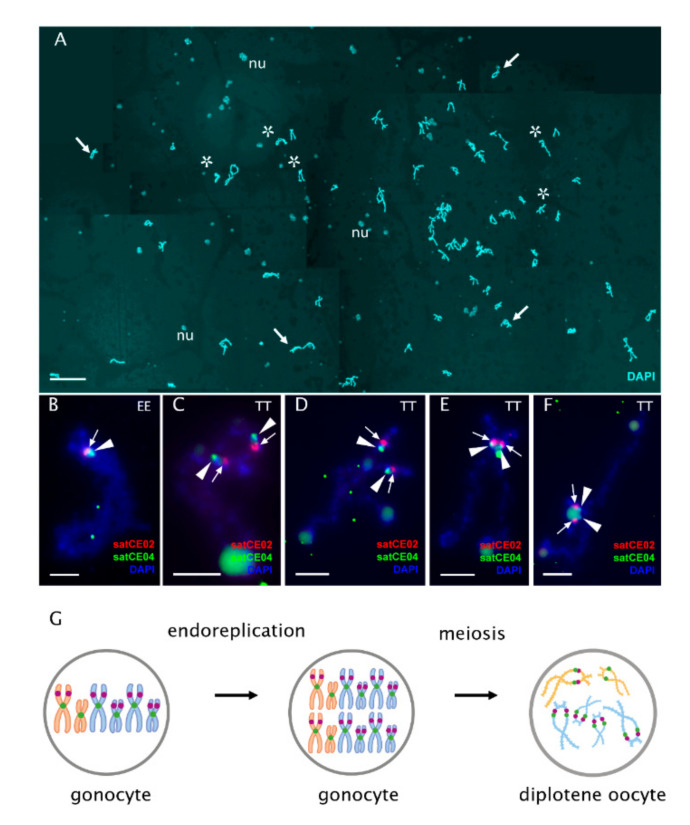
FISH-based identification of bivalents from diplotene oocyte of triploid ETT female. (**A**) Full diplotene chromosomal spread from the individual oocyte including 73 bivalents stained with DAPI (cyan). Thick arrows indicate examples of individual bivalents; nu shows examples of extrachromosomal nucleoli. Since the chromosomal spread from the individual oocyte was large, five images were taken and merged into one. Asterisks indicate enlarged bivalents represented on (**B**–**F**) panels. Scale bar = 50 µm. (**B**–**F**) High-resolution mapping of species polymorphic (satCE02, red; indicated by arrows) and centromeric markers (satCE04, green; indicated by arrowheads) on individual bivalents. Using the combination of two markers and bivalent morphology, a bivalent of *C. elongatoides* (**B**) and 4 bivalents of *C. taenia* (**C**–**F**) were identified. Scale bar = 5 µm. (**G**) Schematic representation of gametogenic pathway in triploid ETT hybrids with the indication of karyotype composition in gonocytes and diplotene oocytes. Only hexaploidy oocytes were observed during diplotene. Premeiotic endoreplication was suggested to form duplicated numbers of chromosomes in diplotene oocytes. *C. elongatoides* chromosomes depicted in orange; *C. taenia* chromosomes depicted in blue. Purple and green marks indicate chromosomes identified by FISH with species polymorphic marker satCE02 and centromeric marker satCE04, respectively.

From a total of 263 observed oocytes during the diplotene stage of meiosis, all possessed the expected number of bivalents/chromosomes (Figure 2 and Figure 3A; Supplementary Table S1; Supplementary Figures S1A and S2A), indicating that a hybrid’s oocytes at the diplotene stage possess a duplicated number of chromosomes compared to somatic cells. We did not observe any sign of abnormal pairing or the presence of multivalents and univalents. Thus, during the diplotene stage of meiosis, diploid hybrids have only tetraploid oocytes, and triploid hybrids have only hexaploid oocytes. To discern whether the bivalents formed by pairing of homologous (ExE and TxT, or NxN) or orthologous (ExT or ExN) chromosomes, we identified bivalents by morphology using previously constructed lampbrush chromosome maps [26]. We found that all bivalents where the aforementioned method was applicable strictly corresponded to the homologous chromosomes emerging after endoreplication, confirming the results of Dedukh et al. (2020; [26]). In addition, we used FISH-based identification of particular chromosomes using species polymorphic satellite markers (satCE02), which is known to occur on two chromosomes in *C. taenia* and a single chromosome in *C. elongatoides* (Supplementary Figure S3C,D,G,H) [43]. Using the satCE02 marker, in diploid hybrids we detected two bivalents of *C. taenia* and one bivalent of *C. elongatoides* while in triploid hybrids we identified four bivalents of *C. taenia* and one bivalent of *C. elongatoides* (Figure 3B–F; Supplementary Figure S1B–D). These observations indicate that diplotene oocytes have their chromosomal sets premeiotically duplicated and that pairing occurs between identical chromosomal copies (Figure 3G; Supplementary Figures S1E and S2B).

### 3.2. Pachytene Falls into Two Types: Those with Duplicated Chromosomes and Proper Bivalents and Those with Few Bivalents and Many Univalents

**Figure 4:**
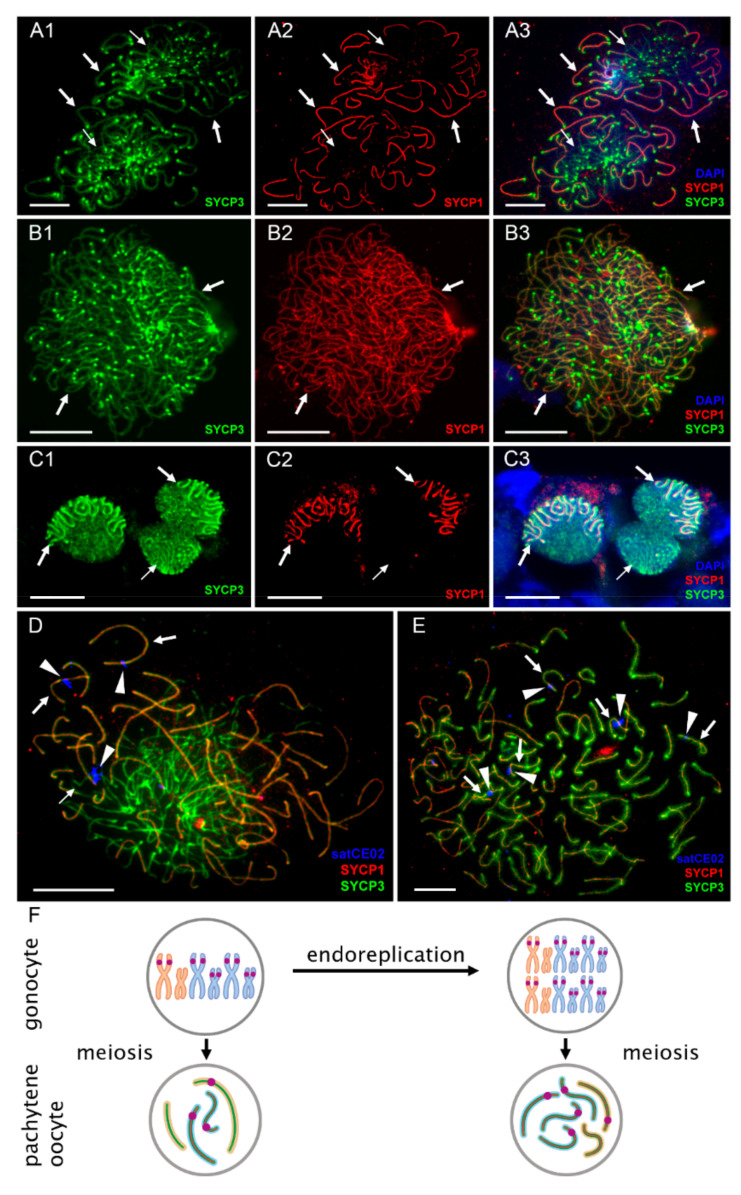
The analysis of pairing and ploidy level in two populations of pachytene oocytes from ovaries of triploid ETT females. Visualization of synaptonemal complexes using immunolabeling with antibodies against its lateral component (SYCP3 protein, green) (**A1**,**B1**,**C1**) and central (SYCP1 protein, red) (**A2**,**B2**,**C2**) components on pachytene chromosomal spreads (**A1**–**B3**) and whole tissues (**C1**–**C3**). Corresponding merged figures presented in (**A3**), (**B3**), (**C3**) also include DAPI staining (blue). Bivalents accumulate both SYCP3 and SYCP1 proteins (indicated by thick arrows in panels (**A1**–**B3**)); univalents accumulate only SYCP3 protein (indicated by thin arrows in panels (**A1**–**B3**)). Triploid pachytene oocyte with both bivalents and univalents (**A1**–**A3**) and hexaploid pachytene oocyte with 73 bivalents (**B1**–**B3**) are presented. Images (**C1**–**C3**) are single confocal sections of 0.6 µm in thickness. Similarly to pachytene spreads, whole mount tissue sections (**C1**–**C3**) show pachytene oocytes with bivalents only (indicated by thick arrow) and cell with both bivalents and univalent (indicated by thin arrow). Mapping of species polymorphic marker (satCE02, blue) (shown by arrowheads) on triploid (**D**) as well as hexaploid pachytene oocytes (**E**). In triploid cell ((**D**), and its schematic representation in F left panel), signals were detected on two bivalents (indicated by thick arrows) and one univalent (indicated by thin arrow). In hexaploid cell ((**E**) and its schematic representation in (**F**) right panel), signals detected on five bivalents (indicated by thick arrows). (**F**) Schematic representation of gametogenic pathways in triploid ETT hybrids with the indication of karyotype composition in gonocytes and pachytene oocytes. During pachytene, triploid oocytes have aberrant pairing with both bivalents and univalents while hexaploid oocytes appeared after premeiotic endoreplication have normal pairing. Purple marks indicate chromosomes identified by FISH with species polymorphic marker satCE02. *C. elongatoides* chromosomes depicted in orange; *C. taenia* chromosomes depicted in blue. Scale bar = 10 µm.

In contrast to diplotenic oocytes, we found a surprising variability in the number of paired chromosomes in oocytes of the pachytene stage. Using immunofluorescence detection of central (SYCP1) and lateral (SYCP3) components of synaptonemal complexes [49,51] we observed that in triploid ETT hybrids, only ~8% (n = 26) of pachytene chromosome spreads had 73 bivalents, as would be expected after premeiotic endoreplication (Figure 2 and Figure 4B1–B3, Supplementary Table S1). The vast majority of their cells possessed only 20–23 bivalents and several unpaired or partially paired chromosomes (Figure 2 and Figure 4A1–A3, Supplementary Table S1). In a widespread triploid EEN clone, only one pachytene cell in one individual out of 40 scored spreads (~1.5%) had duplicated sets of chromosomes (Figure 2, Supplementary Table S1; Supplementary Figure S4A1–B3).

A similar pattern was seen in natural ET diploids, with ~6% (*n* = 24) pachytene spreads containing 49 bivalents and the vast majority of cells showing a combination of 12–15 bivalents, 1–2 multivalents and several univalents (Figure 2; Table S1; Supplementary Figure S5A1–B3). The incidence of cells with proper bivalents during pachytene was even lower in experimental F1 hybrids where only one cell out of 299 showed a signal of 49 properly formed bivalents after endoreplication (Figure 2; Table S1).

The differences between hybrid biotypes (F1 ET, natural ET, natural ETT and natural EEN) in the proportion of duplicated and nonduplicated pachytene cells was highly significant (generalized linear model with binomial error distribution, *p*.val = 13 × 10^−5^). Post hoc tests further showed significant differences among pairs of biotypes containing the comparisons between F1 ET and natural ET (*p*.val = 9 × 10^−5^) or ETT (*p*.val = 39 × 10^−5^). However, no significant differences were observed between pairs containing F1 ET and natural EEN, either between EEN and natural ET or ETT, or between natural ET and ETT. In summary, we may conclude that F1 diploid ET hybrids had a significantly lower proportion of duplicated cells as compared to their natural counterparts, while in EEN the low number of scored cells prevented any clear-cut conclusion from being made.

To verify previous observations on nuclear spreads, we also performed whole mount immunofluorescent staining on pachytene oocytes inside entire gonadal fragments. We did so by investigating the gonads of diploid and triploid hybrid females using the same antibodies against SYCP1 and SYCP3, and similar to previous analyses, we also detected two types of pachytene cells: those with only bivalents and those containing a mixture of univalents and bivalents (Figure 2 and Figure 4C1–C3; Table S1). Interestingly, the analysis of entire gonads indicated that pachytene cells containing only bivalents were not organized in clusters but were surrounded by cells with both bivalents and univalents (Figure 4C1–C3).

### 3.3. Pachytene Oocytes Containing Bivalents and Univalents Do Not Have a Duplicated Genome, While Those with Only Bivalents Do

To discern whether the two cell types in pachytene differ in the number of genomic copies, we determined their ploidy by applying FISH with the species polymorphic satellite marker satCE02 to pachytene chromosomal spreads [43]. We observed that triploid’s pachytene oocytes with exclusively bivalents contained satCE02 signals on five bivalents; four bivalents correspond to *C. taenia* bivalents and one bivalent corresponds to the *C. elongatoides* bivalent (Figure 4E). These results matched our observations of diplotene using the same FISH marker (see above; Figure 3B–F). By contrast, spreads of pachytene oocytes containing a mixture of bivalents and univalents expressed satCE02 signals on only two bivalents and one univalent. These bivalents probably correspond to TT chromosomes (diploid set) and one univalent possibly relating to the E chromosome (haploid set; Figure 4D).

We may therefore conclude that the two types of pachytene cells differ in ploidy in all observed diploid and triploid hybrids. Thus, during pachytene, diploid hybrids have diploid and tetraploid oocytes; triploid hybrids have triploid and hexaploid oocytes. Tetraploid oocytes in diploid hybrids and hexaploidy oocytes in triploid hybrids emerged from gonocytes with duplicated genomes that assure proper pairing as each chromosome has an identical copy to pair with (Figure 4F, right panel; Supplementary Figure S4C, right panel; Supplementary Figure S5C, right panel). However, in triploid hybrids, triploid oocytes (ETT and EEN) have not passed through genome duplication and their bivalents occasionally formed by homologues *C. taenia* (in the case of ETT hybrids) or *C. elongatoides* (in the case of EEN hybrids) chromosomes, while the single copies exist mostly as univalents (Figure 4C, left panel; Supplementary Figure S4C, left panel). In diploid hybrids, diploid oocytes have occasional pairing between *C. elongatoides* and *C. taenia* chromosomes (Supplementary Figure S5C, left panel).

**Figure 5:**
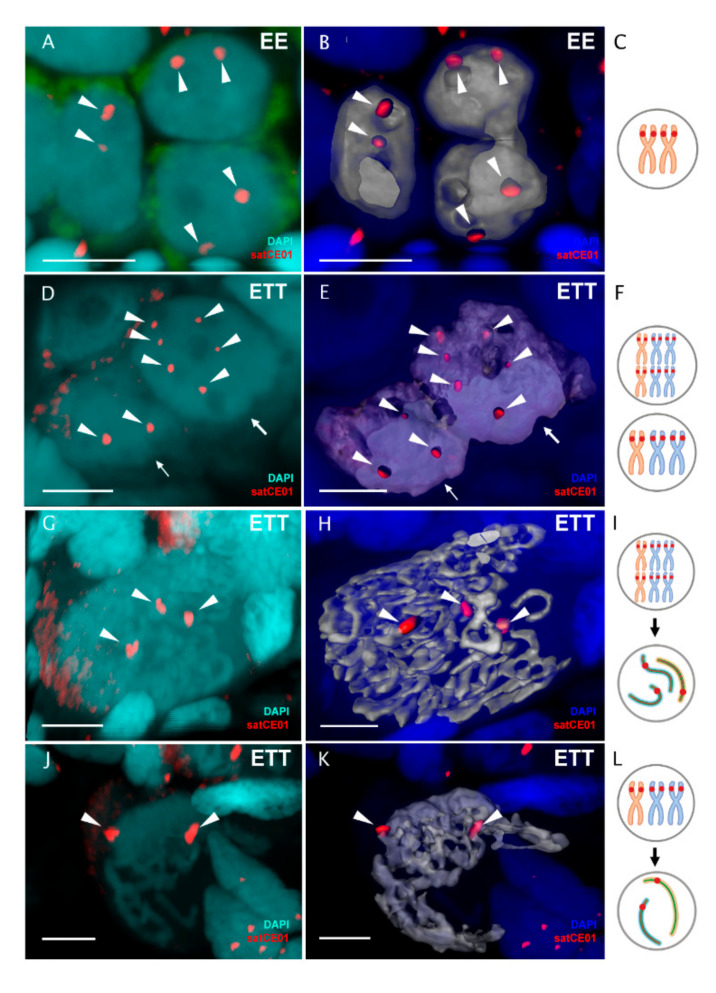
Identification of germ cell ploidy level using FISH with chromosome-specific probe in *C. elongatoides*, and triploid hybrids. Whole-mount FISH with chromosome-specific marker satCE01 (**A**) and corresponding 3D surface reconstruction (**B**) distinguish two chromosomes in germ cells of *C. elongatoides*. Whole-mount FISH with chromosome-specific marker satCE01 (**D**) and corresponding 3D surface reconstruction (**E**) identifies three chromosomes in a germ cell with a nonduplicated genome (triploid cell) (indicated by thin arrow) and six chromosomes in a germ cell with a duplicated genome (hexaploid cell) (indicated by thick arrow) in ovary from triploid ETT hybrid. (**C**,**F**) Schematic representation of chromosomes identified by chromosome-specific marker (red marks) of germ cells on (**A**,**B**,**D**,**E**) panels. *C. elongatoides* chromosomes marked orange and *C. taenia* chromosomes marked blue. Whole-mount FISH with chromosome-specific probe (**G**,**J**) and corresponding 3D surface reconstructions (**H**,**K**) distinguish pachytene oocyte with duplicated genome (**G**,**H**), and pachytene oocyte with nonduplicated genome (**J**,**K**). Schematic representation of bivalents and univalents in pachytene oocyte with duplicated genome (**I**) and in oocyte with nonduplicated genome (**L**) as well as presumptive karyotype composition causing such pairing. Red marks indicate bivalents identified by FISH with chromosome-specific marker satCE01 as well as presumptive karyotype composition in gonocytes. Images (**B**,**E**,**H**,**K**) are 3D reconstructions of natural confocal sections showing the views of germ cells with visualized chromosomes by FISH with chromosome-specific marker. Chromosome-specific marker shown in red (indicated by arrowheads), DAPI stained blue. Scale bar = 5 µm.

Additionally, we examined the ploidy of pachytene oocytes in intact gonads of diploid and triploid hybrid females using whole mount FISH with chromosome-specific probe satCE01 [43]. In the somatic cells of diploid hybrids, this marker identifies one chromosome of *C. taenia* and one chromosome of *C. elongatoides*, while in the somatic cells of triploid ETT hybrids, it determines two chromosomes of *C. taenia* and one chromosome of *C. elongatoides* (Supplementary Figure S3A,B,E,F) [43]. This marker, therefore, allows the distinguishing of the ploidy of pachytene cells only in triploid hybrids but not diploids because pachytene cells of triploid hybrids with two signals would have their genome unduplicated as one signal comes from a bivalent between two TT chromosomes while another signal comes from one E univalent (Figure 5J–L). This was exactly the case in cells with a mixture of bivalents and univalents. By contrast, in the case of pachytene cells with bivalents only, we observed three signals from two TT bivalents and one EE bivalent, suggesting their genome was duplicated (Figure 5G–I). Such analyses confirmed the aforementioned observations on pachytene chromosomal spreads. Moreover, we also discovered that pachytene cells with duplicated genomes do not usually form large clusters but instead occur as individual cells and at most form clusters of 2–4 duplicated cells.

### 3.4. Gonocytes (Germ Cells) Also Occur in Unduplicated and Duplicated Forms within Intact Gonad of Hybrid Females

Finally, we tested whether two ploidy types of cells also exist in the stage of gonocytes. To identify gonocyte cells within the ovarian tissues, we initially applied antibodies against Vasa protein and determined their distinct morphology (Supplementary Figure S6). We subsequently identified the gonocyte’s genome composition using whole mount FISH with chromosome-specific marker (satCE01). In sexual species, we detected two signals per gonial cell (Figure 5A–C). In three triploid fish (ETT) we obtained data on a number of duplicated and nonduplicated cells both at the level of pachytene oocytes as well as gonocytes. We detected on average 89% without genome duplication (cells with 3 signals) and 11% with genome duplication (cells with 6 signals, *n* = 55) (Figure 5D–F; Table S1). Although the ratio of duplicated cells seemed slightly higher among gonocytes, the difference was not significant (generalized linear mixed effect model with individual taken as a random factor and binomial error distribution, *p*-value = 0.12).

## 4. Discussion

### 4.1. Genome Duplication Is Restricted to the Minor Cell Population While the Majority of Cells May Not Proceed beyond Pachytene

**Figure 6:**
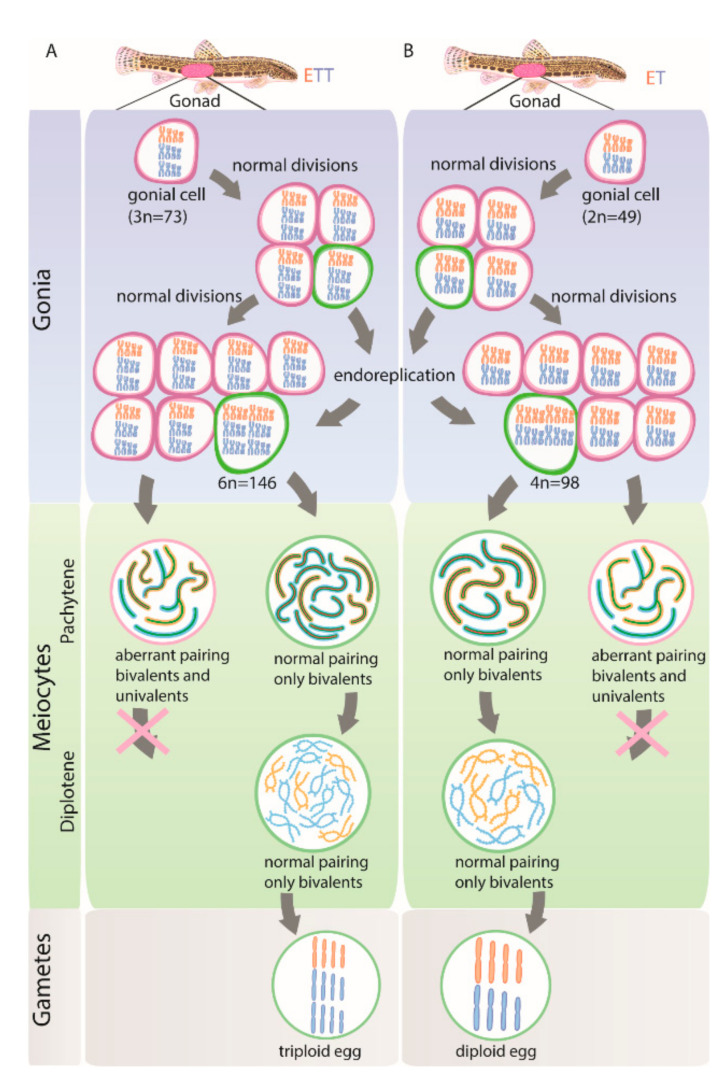
Schematic overview of gametogenic pathway during clonal gametogenesis in triploid ETT (**A**) and diploid ET (**B**) hybrids. Each column shows individuals, gametogenic pathways with the indication of the germ cells, cells in two meiotic stages (pachytene and diplotene) and gametes. E (orange chromosomes), T (blue chromosomes) indicates genomes of both parental species *C. elongatoides* and *C. taenia* correspondingly. Cells with premeiotic genome duplication (green outlines) emerged in individual gonocytes before meiosis. After entering meiosis such cells form 73 bivalents (**A**) or 49 bivalents (**B**) during pachytene and diplotene followed by the formation of triploid (**A**) and diploid (**B**) gametes. Gonocytes with non-duplicated genome (pink outlines) enter meiosis and form univalents and bivalents during pachytene. Such cells cannot proceed beyond pachytene.

The transition from sexual reproduction to asexuality is often triggered by hybridization and many such hybrid asexual organisms are known to undergo premeiotic genome endoreplication to produce clonal gametes [20,22,26,29,30,31,45,52,53]. However, it is largely unknown how widespread this mechanism is within gonial cells of given asexual organisms. To address this, we inspected the genome composition of oocytes during pachytene and diplotene meiotic stages and also of gonial cells in laboratory-produced F1 hybrids as well as in natural diploid and triploid asexuals of the *Cobitis* fish. Surprisingly, premeiotic genome endoreplication was observed in only a minor fraction of the hybrid’s gonocytes (Figure 2 and Figure 6A,B), while the vast majority of gonial cells were unable to duplicate their genomes, causing abruption of pairing and bivalent formation during the pachytene stage (Figure 6A,B). To our knowledge, similar analysis has only been performed in earlier studies, which inferred the rarity of the endoreplication event by examination of the DNA content of gonocytes from parthenogenetic whiptail lizards (from the genus *Aspidoscelis*) and the analysis of meiotic chromosomal spreads in F1 diploid hybrids between two medaka species [37,38,39]. Together with our investigation, such data suggest that even successful natural hybrid asexuals suffer from genome incompatibilities and improper chromosomal pairing, while endoreplication, which restores hybrid fertility by allowing chromosomal pairing between identical chromosomal copies, is a rare event during gonocyte multiplication.

We further discovered that the ratio of duplicated/non-duplicated cells drastically changed between pachytene and diplotene stages. In diplotene, we observed oocytes exclusively with duplicated genomes and proper bivalents without any univalents or aberrant pairing. This suggests that oocytes, which may not form proper bivalents without premeiotic endoduplication, cannot proceed beyond the pachytene stage (Figure 6A,B). Such observation coincides with the well-known “pachytene checkpoint” that involves efficient DSB repair machinery and elements controlling synapsis [54,55]. As these pathways are highly conserved between yeast, nematodes, insects and mammals [56,57,58,59], we expect that similar processes take place in *Cobitis* hybrid females and probably in other asexuals too. We may thus propose that the pachytene checkpoint prevents the progression of non-duplicated oocytes with incompletely paired chromosomes beyond pachytene, potentially causing the death of such cells, thereby preventing the growth and formation of aberrant gametes.

### 4.2. Sex-Specific Differences in Meiotic Checkpoints and Stringency of Pairing Chromosomal Control

Our observations also offer an interesting insight into the sex-specific differences in gametogenic control when compared to previous analysis of hybrid males. Namely, during meiosis of diploid and triploid male hybrids between *C. elongatoides* and *C. taenia*, Dedukh and co-authors [26] observed aberrant spermatocytes with univalents and multivalents, both in the pachytene stage and in the metaphase of meiosis I. Thus, contrary to hybrid females, male pachytene spermatocytes with aberrant pairing are fully or partially able to bypass the pachytene checkpoint [26]. Nevertheless, such cells seem to become trapped during the spindle assembly checkpoint acting during metaphase 1 [60,61]. Spindle assembly checkpoint machinery assesses the stringency of the spindle in each bivalent and allows progression beyond metaphase only when all bivalents are correctly arranged [60,61]. Thus, meiotic progression in male hybrids is prevented at later stages by the failure of the equal stringency from the spindle caused by univalents [62,63].

Differences between sexes in meiotic checkpoints were also observed in other organisms, but the patterns somewhat contradict each other [61,64,65]. For example, the hybrids between medaka fish *Oryzias latipes* × *O. curvinotus* showed similar patterns to *Cobitis* since oocytes with aberrantly paired chromosomes could not proceed beyond pachytene, while spermatocytes with aberrant pairing did not disrupt meiotic prophase but also progressed to metaphase of meiosis I [38,66]. Such abruption of meiosis is probably caused by the abnormal alignment of bivalents at the spindle suggesting that meiotic progression is blocked due to the inability to pass through the spindle assembly checkpoint [38,60,61,62,63,66]. By contrast, in asexual triploid hybrid females from *Misgurnus,* the sister genus to *Cobitis,* oocytes with univalents and bivalents apparently proceed to diplotene [67]. Moreover, they also overcome the spindle assembly checkpoint, as univalents do not attach to the spindle and are lost during the anaphase of meiosis I while chromosomes forming bivalents segregate normally [67]. Mammals appear to be different from *Cobitis* and other aforementioned cases as the defects in the DSB repair system or synapsis typically lead to cell death during the pachytene stage of mammalian spermatocytes, while in other species, defective oocytes often proceed through both meiotic divisions [68,69]. Taken together, we suggest that having the capacity to overcome different gametogenic pathways (for instance, pachytene or spindle assembly checkpoints) is crucial for the reproduction of asexual hybrid females.

### 4.3. Initiation of Premeiotic Genome Duplication

A very efficient mechanism to alleviate problems in orthologue pairing and simultaneously gain clonal reproduction is premeiotic genome duplication (this study; [26,38]). Yet, despite its widespread occurrence across major animal and plant taxa, it remains a surprisingly understudied phenomenon with very little known about when the duplication event occurs or how it is triggered.

Our observations can deliver at least some information regarding this. Fish oogonia are able to divide throughout the lifetime of the animal and are maintained as gonial stem cells that can subsequently enter meiosis [70,71,72]. During their proliferation, the gonocytes actively divide and form clusters (“nests”), which contain all descendants of a single progenitor germ cell. If genome endoreplication occurs during the early proliferation stage of gonocytes, large clusters of exclusively cells with duplicated genomes would be expected. However, our analysis of pachytene cells and gonocytes found no evidence of such clusters as we mostly observed individual nuclei with duplicated chromosomal sets, or rarely small groups of 2–4 endoreplicated cells. We may thus conclude that endoreplication occurs in gonocytes just before entering meiosis or occasionally 1–2 divisions before such an event.

In *Misgurnus* loaches, Yoshikawa and co-authors [73] described a different timing of endoreplication in the gonocytes of hybrid males hormonally reversed into females. They found that endoreplication occurred in sex-reverted hybrid males already in A-type spermatogonia, which somewhat contradicts our observation in *Cobitis* females, since such spermatogonia still have several mitotic divisions before turning into B-type spermatogonia and thus, entering meiosis. Such a comparison suggests that premeiotic endoreplication may proceed differently even in closely related organisms such as *Cobitis* and *Misgurnus*. Alternatively, the initiation of endoreplication may be caused by methodological approaches and these contrasting results could be obtained for instance by the developmental shock associated with sex-reversal as suggested by Yoshikawa et al. [73].

While detecting the timing of endoreplication is challenging, it is even harder to identify the causal triggers initiating this process in asexual hybrids. Interestingly, however, three independent studies on unrelated organisms documented that endoreplication affects only a rather minor fraction of gonial cells in asexual hybrids (such as lizards and at least two fish species (this study; [37,38,39])). This may indicate that some similarities exist in the underlying mechanisms causing this aberration. We therefore believe that understanding of the mechanisms causing genome duplication in germ cells of asexual hybrids can be improved based on studies implemented on polyploid/aneuploid cells observed in a variety of organisms, e.g., flies, mice, humans and the zebrafish [74,75,76,77]. For instance, it has been documented that endopolyploidy affecting some cells or tissues emerge during development or under stress conditions [78,79,80]. In different organisms, several cell cycle regulators were found to be responsible for the switch from normal mitosis to endomitosis/endoreplication cycles, specifically when they are downregulated or their function is affected [81,82,83,84]. Among these regulators, Cyclin A/Cyclin dependent kinase 1 and Aurora B kinase play a crucial role [81,82,83,84,85,86]. Moreover, in their elegant work, Rotelli and co-authors (Rotelli et al., 2019) showed the link between the concentration of CDC1 and AurB to the stringency of effects on the cell cycle. Knockdown of CDK 1 or AurB in human cancer cells and *Drosophila* embryos inhibited chromosome segregation and cytokinesis causing G-S cycles (endoreplication), whereas a hypomorph resulted in successful chromosome segregation but the failure of cytokinesis (endomitosis) [84,87]. We may therefore hypothesize that in hybrids some proteins involved in the Cyclin/Cdk1—AurB pathway can be translated from genomes of different parental species forming enzyme heterocomplexes with a decreased function as compared to proteins composed of products from the same species. Such heterocomplexes may then lead to a decreased level of AurB kinase causing a round of endoreplication/endomitosis in individual gonocytes of hybrid females.

### 4.4. Implications for Ecology and Evolution of Asexual Organisms

The emergence of gametogenic aberrations leading to asexuality was suggested to be either directly triggered by hybridization [88] or emerge as a result of preconditions in sexual species with hybridization just accelerating this process [89,90]. One way or another, asexual reproduction provides a considerable short-term advantage by avoiding the cost of male offspring and, all else being equal, asexuals should quickly outcompete their sexual counterparts. In reality, however, sexual and asexual counterparts differ by many traits. For example, asexuality is often linked to polyploidy, which modifies the metabolic rate [91], or size-selective predation mortality [92,93] when a larger size of polyploid offspring allows faster escape from natural predators [94]. In any case, the extinction of sexuals would leave sperm-dependent asexuals like gynogens without a source of sperm and hence some mechanisms are clearly necessary in order to stabilize sexual–asexual coexistence. These usually assume some behavioral adaptations linked to mate choice [95,96,97], but it has been suggested that increased mortality or sterility of asexual individuals may be beneficial for gynogenetic populations since it would compensate the inherent demographic advantage of asexuality and stabilize their coexistence with sexual species [98,99]. In the *Cobitis* hybrid complex, Bobyrev et al. [98] and Juchno and Boroń [44,100] reported an approximately 50% reduction in fecundity in triploid *elongatoides*-*taenia* hybrid females compared to the *C. taenia* parental species indicating the potential existence of such compensation in natural populations. However, the reported drop in fecundity is far lower than our observation of failure in pachytene oocytes, suggesting that some cellular mechanism(s) compensate the dramatic loss at the pachytene checkpoint.

Although possibly advantageous at population level, such a high rate of gametogenic failure is disadvantageous for individuals and it is likely that short-term selection should favor those hybrid clones that minimize the negative effect of genomic incompatibilities. Our data support such a hypothesis as we found a significantly higher rate of endoreduplication in successfully established natural clones as compared to experimental F1 clones originated from crossings of randomly selected parents. Thus, although initiation of asexuality is achieved frequently by hybridization of *C. elongatoides* with other species ([14], this study), interclonal selection appears to favor hybrid clones that originated from particular combinations of parental genomes allowing the highest rates of endoreduplication. It is also possible that after their formation some clones evolve to further ameliorate such capability. However, the most widespread and oldest *Cobitis* clone (EEN) has a significantly higher fecundity compared to relatively younger ET and ETT lineages that originated in Central Europe during the Holocene. Earlier, we found no evidence for any higher rate of endoreduplication in this successful lineage, suggesting that selection may possibly also operate on later stages of gametogenic processes [101].

## 5. Conclusions

In conclusion, asexuality and hybrid sterility are intimately intertwined phenomena and transitions from sexual reproduction to asexuality may bring considerable costs, especially in hybrid taxa that face significant problems with genome incompatibilities, potentially greatly reducing their reproductivity. Nevertheless, asexuals may establish very successful and persistent lineages even with such an expense, possibly helping them to maintain a stable coexistence with sexual hosts. Our study also stresses the importance of using several approaches experimentally, as demonstrated by the striking differences in cell ploidy detected in pachytene and diplotene stages that were detected by different experimental means. It should be noted that taking into account only one type of analysis could lead to misinterpretation of the results. Finally, it also appears of crucial importance to collect detailed insights into gametogenic pathways of various asexuals in order to understand the mechanistic interlink between hybridization, sterility and asexuality.

## Data Availability

The authors state that all data necessary for confirming the conclusions presented in the article are represented fully within the article.

## Supplementary Materials

The following are available online at https://www.mdpi.com/article/10.3390/ijms222212117/s1.
