# Supplementary material for: Challenges and Costs of Asexuality: Variation in Premeiotic Genome Duplication in Gynogenetic Hybrids from Cobitis taenia Complex
Source: Int J Mol Sci. 2021 Nov 9;22(22):12117. doi: 10.3390/ijms222212117 (PMC8622741; doi:10.3390/ijms222212117)
Supplement: Supplementary file 1 [file ijms-22-12117-s001.zip › ijms-1421835-supplementary.pdf]

Supplementary files

Challenges and costs of asexuality: Variation in premeiotic genome duplication in gynogenetic hybrids from *Cobitis taenia* complex.

Dedukh D., Marta A., Janko K.

Figure S1. FISH based identification of bivalents from diplotene oocyte of diploid ET female. (A) Full diplotene chromosomal spread including 49 bivalents stained with DAPI (cyan). Thick arrows indicated examples of individual bivalents; nu shows examples of extrachromosomal nucleoli. Since the chromosomal spread was large, eight images were taken and merged into one. Asterisks indicate enlarged bivalents represented on B-D panel. (B-D) Scale bar = 50 μm.

(B-D) High-resolution mapping of species polymorphic (satCE02, red; indicated by arrows) and centromeric (satCE04, green; indicated by arrowheads) markers on individual bivalents during diplotene. Using the combination of two markers and bivalents morphology, Bivalent of *C. elongatoides* (B) and two bivalents of *C. taenia* (C,D) were identified. Scale bar = 5 μm.

(E) Schematic representation of gametogenic pathway in diploid ET hybrids with the indication of karyotype composition in gonocytes and diplotene oocytes. Only tetraploid oocytes were observed during diplotene. Premeiotic endoreplication was suggested to form duplicated number of chromosomes in diplotene oocytes. *C. elongatoides* chromosomes depicted in orange; *C. taenia* chromosomes depicted in blue. Purple and green marks indicate chromosomes identified by FISH with species polymorphic marker satCE02 and centromeric marker satCE04 correspondingly.

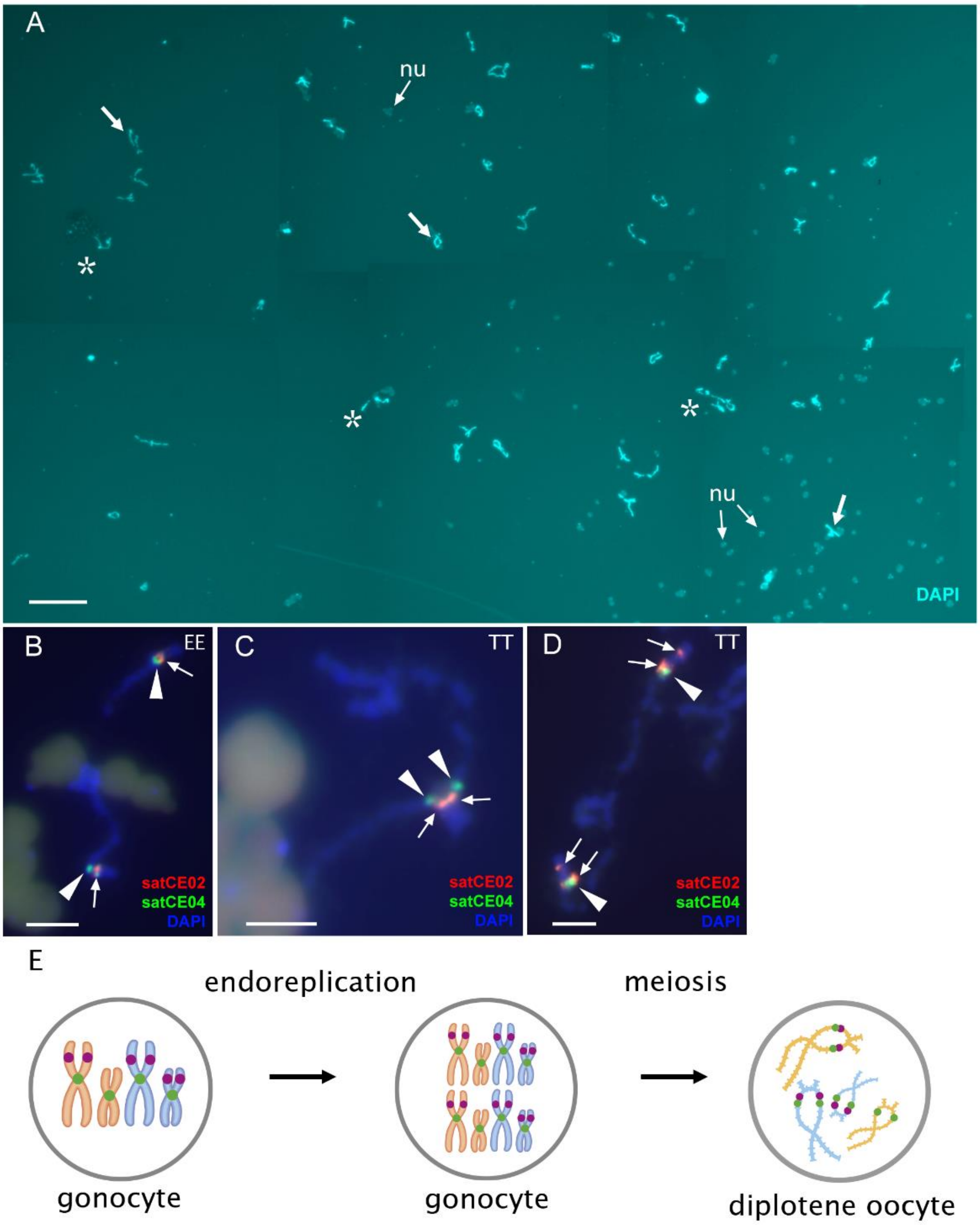
A
nu
nu
DAPI
B
EE
satCE02
satCE04
DAPI
C
TT
satCE02
satCE04
DAPI
D
TT
satCE02
satCE04
DAPI
E
endoreplication
meiosis
gonocyte
gonocyte
diplotene oocyte

Figure S2. Diplotene oocyte chromosomal spread from ovary of EEN female. (A) Full diplotene chromosomal spread including 75 bivalents stained with DAPI (cyan). Thick arrows indicated examples of individual bivalents; nu shows examples of extrachromosomal nucleoli. Since the chromosomal spread was large, 12 images were taken and merged into one. Scale bar = 50μm. (B) Schematic representation of gametogenic pathway in triploid EEN hybrids with the indication of karyotype composition in gonocytes and diplotene oocytes. Only hexaploid oocytes were observed during diplotene. Premeiotic endoreplication was suggested to form duplicated number of chromosomes in diplotene oocytes. *C. elongatoides* chromosomes depicted in orange; *C. tanaitica* chromosomes depicted in blue.

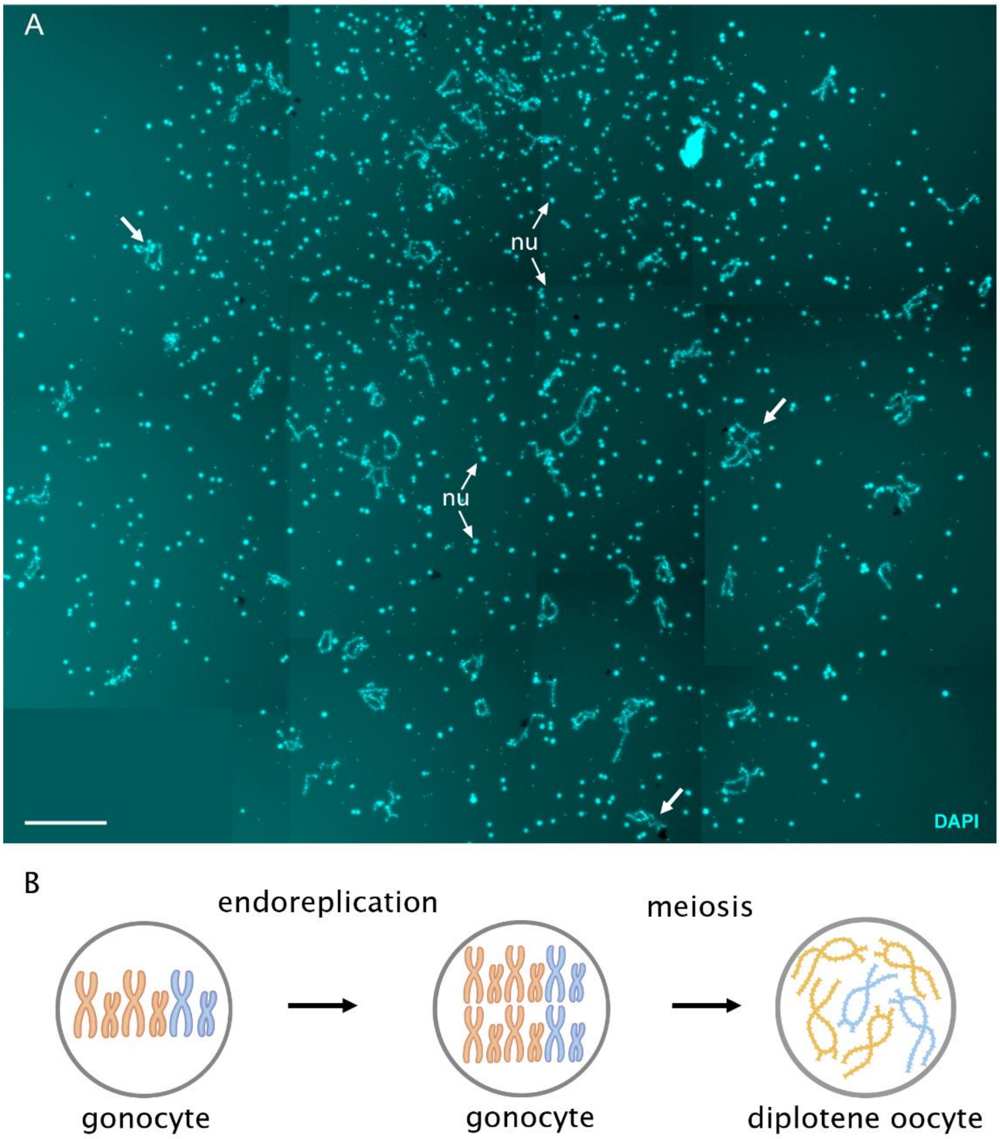
A
nu
nu
DAPI
B
endoreplication
meiosis
gonocyte
gonocyte
diplotene oocyte

Figure S3. Mapping of satDNA markers on mitotic metaphase chromosomes of *C. taenia* (A, C) and *C. elongatoides* (E, G). Arrows indicate the localization of chromosome specific marker SatCE01 (A, E), and species polymorphic marker SatCE02 (C, G) on mitotic metaphase chromosomes. Schematic representation of chromosomes distinguished by satDNA markers within karyotype of *C. elongatoides* (F, H; marked orange) and *C. taenia* (B, D; marked blue). Red marks indicate chromosome specific marker (SatCE01); purple marks indicate species polymorphic marker (Sat CE02).

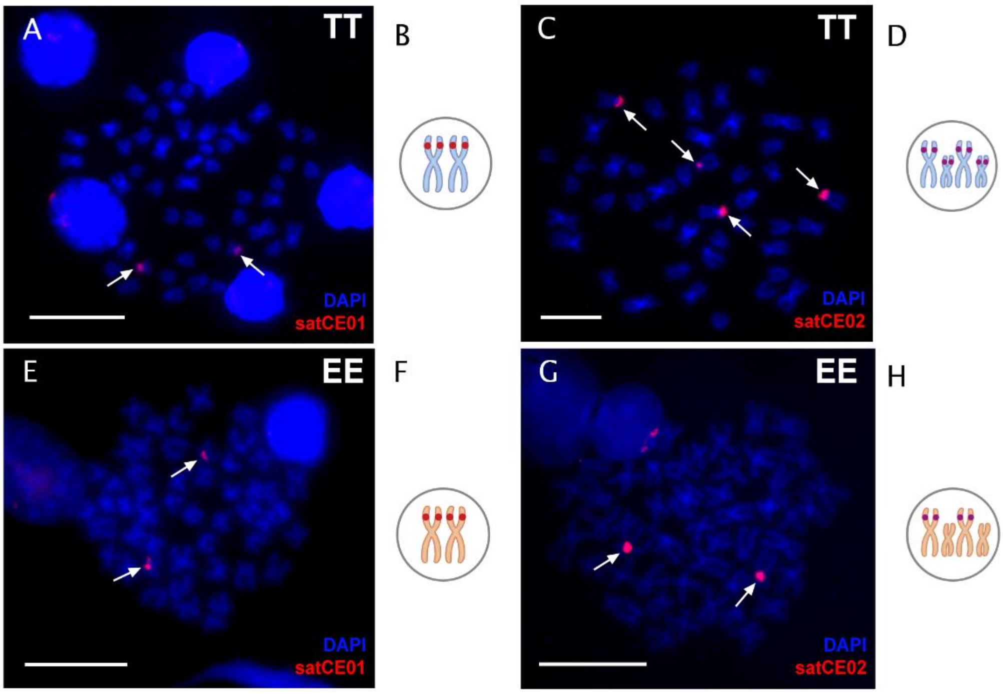
A
TT
B
C
TT
D
DAPI
satCE01
DAPI
satCE02
E
EE
F
G
EE
H
DAPI
satCE01
DAPI
satCE02

Figure S4 Meiotic oocytes at pachytene stage from ovaries of triploid EEN females. Visualization of synaptonemal complexes using immunolabeling with antibodies against its lateral component (SYCP3 protein, green) (A1, B1) and central component (SYCP1 protein, red) (A2, B2) on pachytene chromosomal spreads. Bivalents show both SYCP3 and SYCP1 (shown by thick arrows) localization while univalents exhibit only SYCP3 staining (shown by thin arrows). (C) Schematic representation of gametogenic pathways which result in bivalent formation in two populations of pachytene cells. Karyotype composition in gonocytes and pachytene oocytes with *C. elongatoides* (orange) and *C. tanaitica* (blue) chromosomes. Scale bar = 10μm.

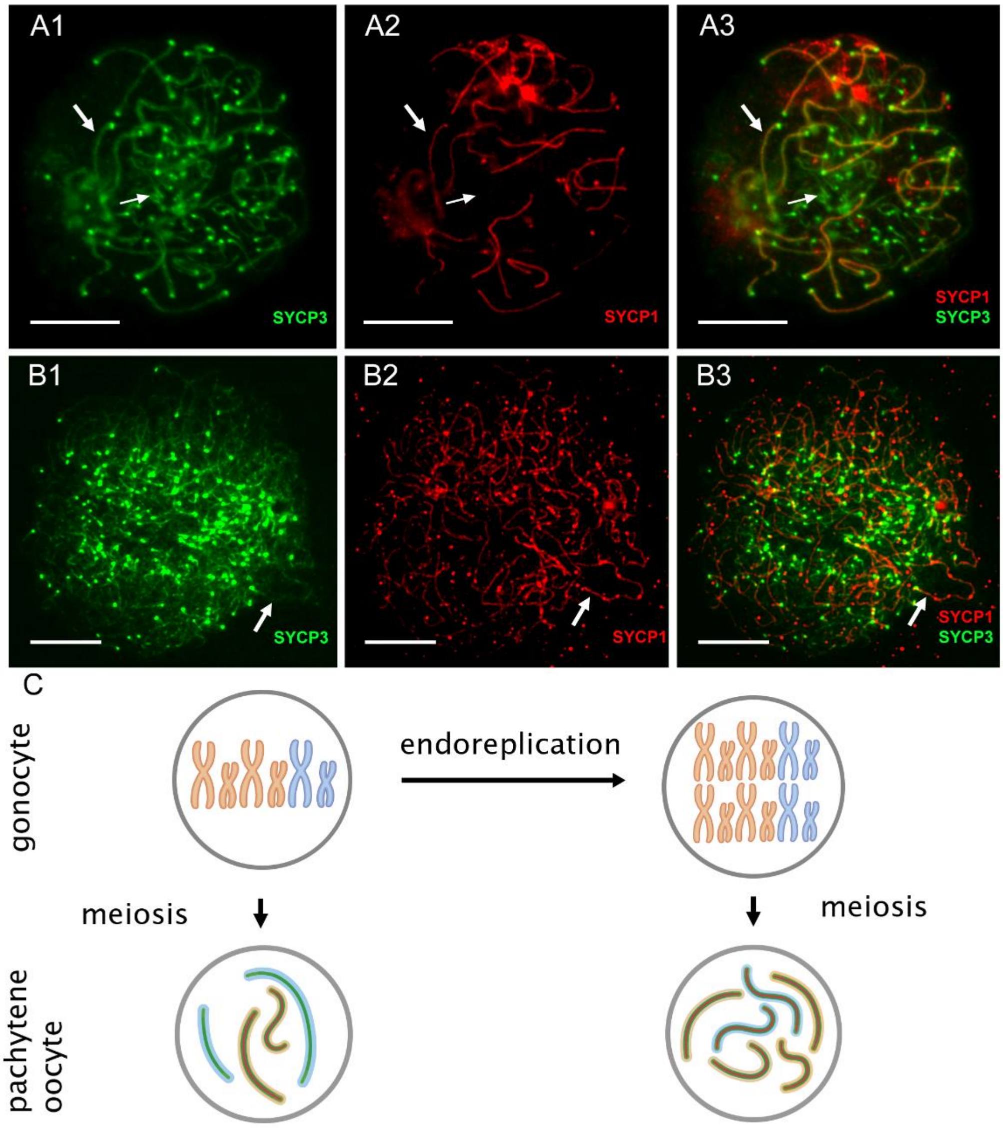
A1
A2
A3
SYCP3
SYCP1
SYCP1
SYCP3
B1
B2
B3
SYCP3
SYCP1
SYCP1
SYCP3
C
gonocyte
endoreplication
meiosis
meiosis
pachytene
oocyte

Figure S5 Meiotic oocytes at pachytene stage from ovaries of diploid ET females. Visualization of synaptonemal complexes using immunolabeling with antibodies against its lateral component (SYCP3 protein, green) (A1, B1, C1) and central component (SYCP1 protein, red) (A2, B2, C2) on pachytene chromosomal spreads. Diploid (A1-A3) and tetraploid (B1-B3) oocytes are detected during pachytene. Bivalents show both SYCP3 and SYCP1 localization (indicated by thick arrows) while univalents exhibit only SYCP3 staining (indicated by thin arrows). (C) Schematic representation of gametogenic pathways in diploid ET hybrids with the indication of karyotype composition in gonocytes and pachytene oocytes. During pachytene, oocytes with two ploidy level were observed: diploid oocytes with aberrant pairing and tetraploid oocytes with normal pairing. Tetraploid oocytes likely emerged after premeiotic genome duplication. *C. elongatoides* chromosomes depicted in orange; *C. taenia* chromosomes depicted in blue. Scale bar = 10μm.

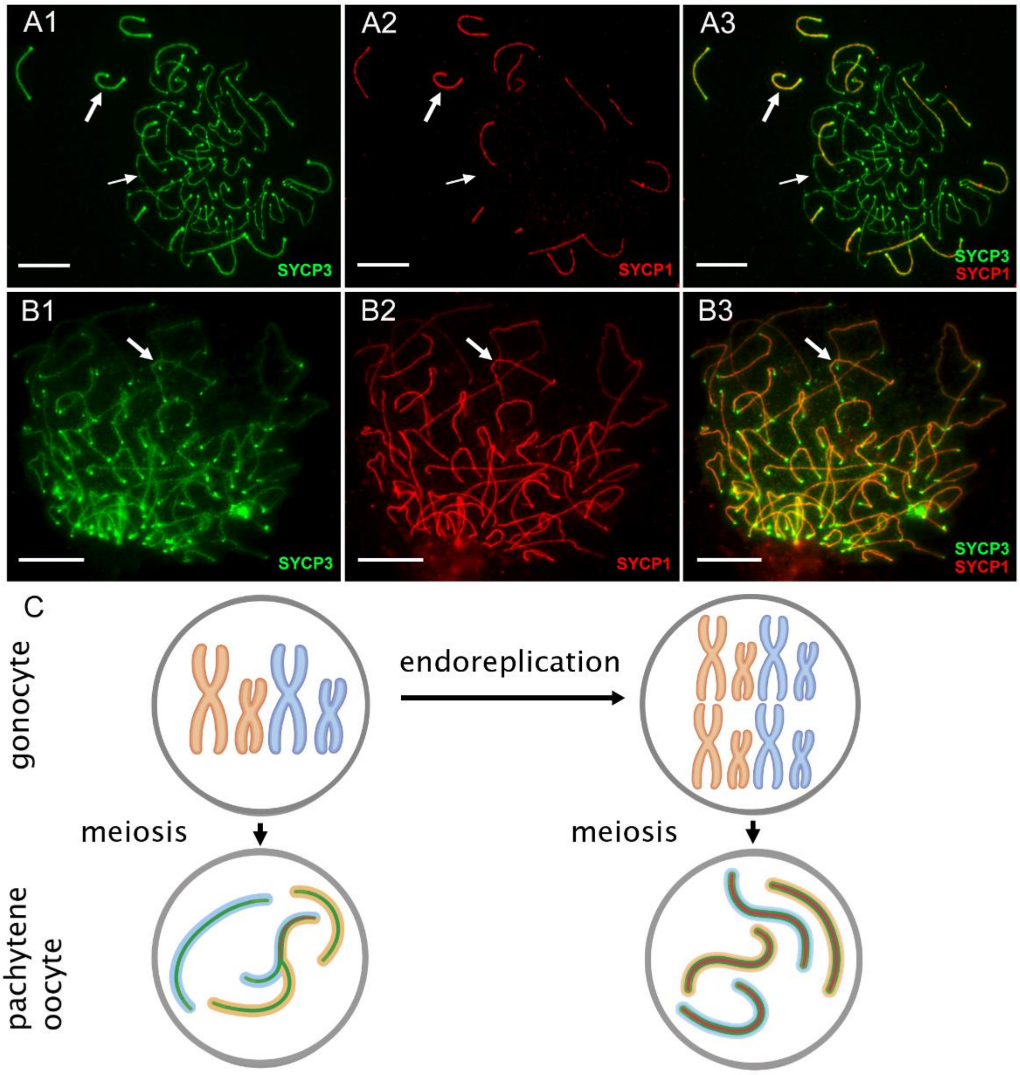

A1
SYCP3
A2
SYCP1
A3
SYCP3
SYCP1
B1
SYCP3
B2
SYCP1
B3
SYCP3
SYCP1
C
gonocyte
endoreplication
meiosis
meiosis
pachytene oocyte

Figure S6 Gonadal microanatomy in triploid ETT hybrid female. Whole-mount immunofluorescent staining with antibodies against Vasa protein (red) allows germ cells (G) identification. According to the morphology of gonads, several cell types can be identified: S, somatic cells; F, follicular cells; G, germ cells; D, diplotene oocyte. DAPI is visualizing chromatin (cyan). Scale bars= 10 µm.

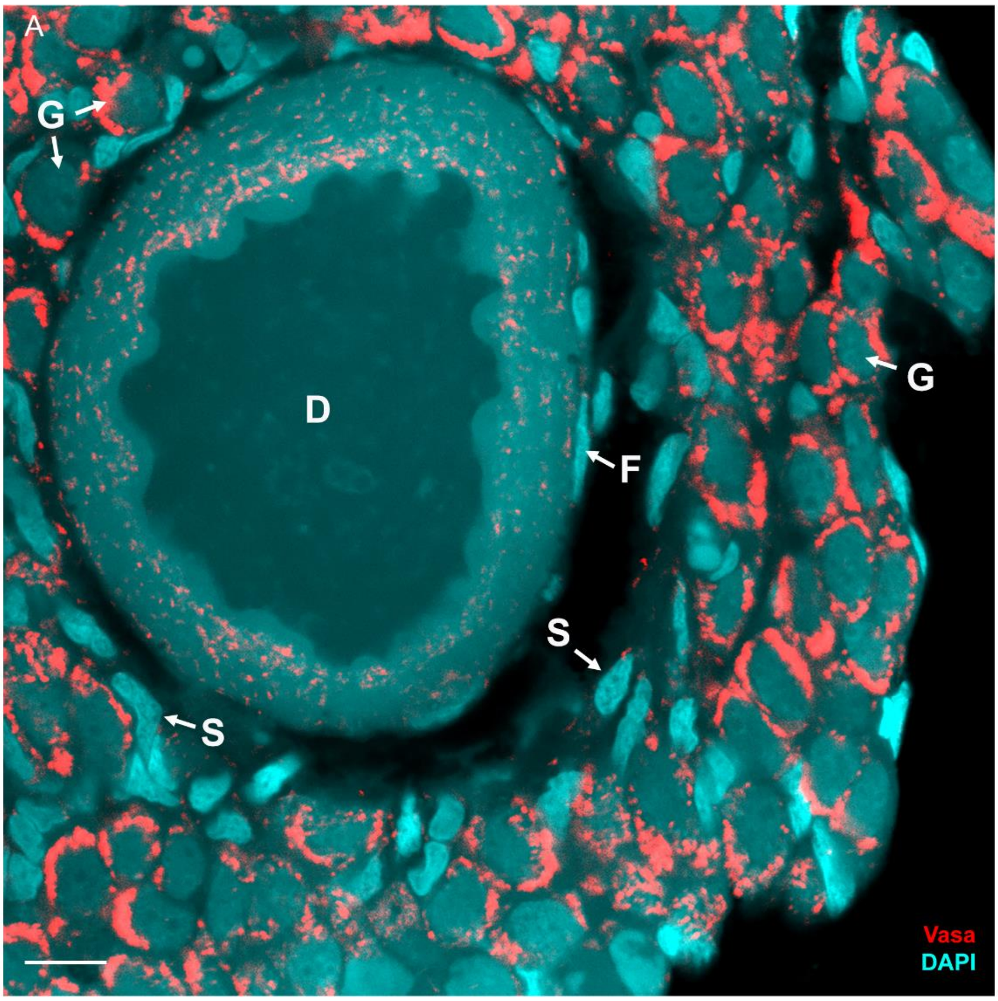
A
G
G
D
F
S
S
Vasa
DAPI

Supplementary Table S1. List of hybrid individuals from *Cobitis* genus used in the study with the indication of cells with duplicated and not duplicated genomes.

#	Individuals	Genotype	pachytene spreads		diplotene spreads		IF pachytenes confocal		FISH pachytenes confocal		FISH germ cells confocal	
			Number of cells with not duplicated genome	Number of cells with duplicated genome	Number of cells with not duplicated genome	Number of cells with duplicated genome	Number of cells with not duplicated genome	Number of cells with duplicated genome	Number of cells with not duplicated genome	Number of cells with duplicated genome	Number of cells with not duplicated genome	Number of cells with duplicated genome
			pachytene spreads		diplotene spreads		IF pachytenes confocal		FISH pachytenes confocal		FISH germ cells confocal	
1	ETT 1	ETT	15	0	N/A	N/A						
2	ETT 2	ETT	32	1	N/A	N/A						
3	ETT 3	ETT	N/A	N/A	0	17						
4	ETT 4	ETT	17	2	0	15						
5	ETT 5	ETT	24	0	0	31						
6	ETT 6	ETT	31	2	N/A	N/A	N/A	N/A	40	4	152	8
7	ETT 7	ETT	19	0	N/A	N/A	62	6	N/A	N/A	N/A	N/A
8	ETT 8	ETT	7	0	N/A	N/A	18	4	32	3	237	25
9	ETT 9	ETT	N/A	N/A	no	32	N/A	N/A	26	4	95	22
1	ET 1	ET	11	0	0	27						
2	ET 2	ET	44	2	0	35						
3	ET 3	ET	N/A	N/A	0	18						
4	ET 4	ET	N/A	N/A	0	18					7	1
5	ET 5	ET	33	11	N/A	N/A						
6	ET 6	ET	19	1	N/A	N/A						
7	ET 7	ET	46	7	N/A	N/A						
8	ET 8	ET	154	0	N/A	N/A						
9	ET 9	ET	11	1	N/A	N/A						
10	ET 10	ET	53	2	N/A	N/A						
11	ET 11	ET	9	0	N/A	N/A						
1	F1_ET 1	F1_ET	51	0	N/A	N/A						
2	F1_ET 2	F1_ET	43	0	N/A	N/A						
3	F1_ET 3	F1_ET	108	1	N/A	N/A						
4	F1_ET 4	F1_ET	21	0	N/A	N/A						
5	F1_ET 5	F1_ET	N/A	N/A	0	15						

6	F1_ET	F1_TE	3	0	N/A	N/A							
7	F1_ET	F1_TE	72	0	N/A	N/A							
8	F1_ET	F1_TE	N/A	N/A	0	9							

1	EEN1	EEN	N/A	N/A	0	31							
2	EEN2	EEN	N/A	N/A	0	15							
3	EEN3	EEN	31	1	N/A	N/A							
4	EEN4	EEN	8	0	N/A	N/A							
